# The Effectiveness and Safety of Exenatide Versus Metformin in Patients with Polycystic Ovary Syndrome: A Meta-Analysis of Randomized Controlled Trials

**DOI:** 10.1007/s43032-023-01222-y

**Published:** 2023-03-31

**Authors:** Zheng-Rong Ye, Can-Qun Yan, Nianchun Liao, Si-Hua Wen

**Affiliations:** 1 Department of Endocrinology, The Third People’s Hospital of Yongzhou, Yongzhou, China; 2 Department of Endocrinology, The Second Affiliated Hospital of University of South China, Hengyang, China; 3 Department of Health Management Center, The Second Affiliated Hospital of University of South China, Hengyang, China; 4 Department of Obstetrics and Gynecology, Changsha Central Hospital, Changsha, China; 5 Department of Abdominal Surgery, The Third People’s Hospital of Yongzhou, Yongzhou, China

**Keywords:** Exenatide, Metformin, Obesity, Reproductive function, Polycystic ovary syndrome

## Abstract

Polycystic ovary syndrome (PCOS) is an endocrine disorder that affects women of childbearing age, resulting in reproductive dysfunction, hyperinsulinemia, and obesity. While several drugs are currently approved for use in these patients, their relative effectiveness remains controversial. The purpose of this meta-analysis was to evaluate the reproductive efficacy and safety of exenatide, a glucagon-like peptide-1 receptor agonist, versus metformin, an insulin sensitizer, in the treatment of patients with PCOS. Nine randomized controlled trials (RCTs) were included, comprising 785 PCOS patients, of whom 385 received exenatide and 400 received metformin. Compared with metformin, exenatide was significantly more effective in treating these patients, as demonstrated by increased pregnancy rate (relative risk (RR) = 1.93, 95% confidence interval (CI) 1.28 to 2.92, P = 0.002), greater ovulation rate (RR = 1.41, 95% CI 1.11 to 1.80, P = 0.004), decreased body mass index (mean difference =  − 1.72 kg/m^2^, 95% CI − 2.27 to − 1.18, P = 0.00001), and improved insulin resistance (standard mean difference =  − 0.62, 95% CI − 0.91 to − 0.33, P < 0.0001). There was no significant difference in the occurrence of adverse events (gastrointestinal reactions, hypoglycemia, etc.) between the two therapies. However, given the moderate to high quality and possible bias of the included studies, the available evidence is inconclusive. More high-quality studies are needed to assess the effects of exenatide in order to provide stronger evidence for its use in this patient population.

**Supplementary Information:**

The online version contains supplementary material available at 10.1007/s43032-023-01222-y.

## Introduction

Polycystic ovary syndrome (PCOS) is an endocrine disease that is mainly characterized by androgen excess, as well as reproductive and metabolic dysfunction [1]. It affects approximately 6%–10% of pre-menopausal women worldwide [2–4]. PCOS typically manifests as impaired ovulation and hyperandrogenism, as the ovaries of PCOS patients show pronounced over-synthesis of steroid hormones compared with normal follicular membrane cells. The most prominent clinical features include reproductive dysfunction, anovulation, and disrupted menstruation [5, 6]. Additional clinical features include hyperinsulinemia, marked insulin resistance [7] and obesity, which interact with each other to aggravate disease progression, as well as hirsutism and/or acne [8]. PCOS not only presents with infertility, but also increases the risk of spontaneous abortion, congenital fetal disease, and obstetric complications in patients who do become pregnant [9]. Furthermore, PCOS has lasting impacts far beyond childbearing age and can influence many aspects of women’s overall health, as it is associated with an increased risk of developing metabolic syndrome, anxiety and depression, and endometrial cancer [10–12].

The core objectives when treating patients with PCOS include improving reproductive system function, decreasing insulin resistance, treating symptoms caused by androgen excess, reducing the risk of cardiovascular complications, and promoting weight loss. Because the pathogenesis of PCOS is still unclear, lifestyle intervention is the first choice for treatment, and the main focus is weight loss, as this is a crucial factor affecting pregnancy outcomes [13]. As many as 74% of patients with PCOS are classified as obese [14]. This obesity is usually associated with hyperinsulinemia, followed by increased ovarian androgen secretion [15], which in turn causes visceral fat deposition, aggravating insulin resistance and further increasing androgen secretion due to elevated insulin levels [16]. These changes are also important causes of ovulation disorders and abnormal menstruation.

In the past, metformin (MET) has been recommended as the first choice for weight loss in patients with PCOS [17]. MET is an insulin sensitizer that reduces insulin levels and improves insulin receptor activity [18], resulting in decreased insulin resistance, lower androgen levels, and improved weight control [19, 20]. In addition to these effects, MET significantly increases ovulation rate compared with placebo [21, 22]. However, evidence regarding its efficacy in optimizing both fertility and pregnancy outcomes is inconclusive [23], and treatment with MET may not be sufficient for addressing reproductive dysfunction in patients with PCOS [24]. Indeed, a recent meta-analysis showed that treatment with MET resulted in very limited improvement in pregnancy and live birth rates compared with placebo [25]. In addition, there are many contraindications to the use of MET, with the most serious potential adverse reaction being lactic acidosis. Thus, treatment of PCOS with MET remains controversial.

In recent years, glucagon-like peptide-1 receptor agonists (GLP-1 RAs) have attracted attention as a new option for PCOS treatment, and are currently recommended by the European Society of Endocrinology for treating this patient population [26]. Among the commercially available GLP-1 RAs, only exenatide (EX) and liraglutide have been recommended for PCOS [27], and so far few randomized controlled trials (RCTs) have shown that liraglutide improves ovulation or pregnancy outcomes, making EX more promising for clinical application. EX, a gut-derived incretin hormone that enhances insulin sensitivity, reduces blood glucose and insulin levels. Moreover, it can inhibit gastric emptying, thus reducing appetite and body weight [28, 29]. In addition, a study performed in rats showed that EX significantly improved endocrine and reproductive status; androgen secretion, body weight, and HOMA-IR were significantly decreased in the rats treated with EX compared with the control group, which may have been related to the increased expression of AMPKα and SIRT11 [30].

Recent studies have suggested that EX provides more adequate control of PCOS symptoms than MET [26, 27, 31, 32], although whether EX improves ovulation and pregnancy rates, as well as whether it has a similar safety profile to MET, is still controversial. While several RCTs have been carried out to answer these important questions, most of them were underpowered to provide a robust and clinically applicable conclusion. Therefore, the aim of this study was to perform a meta-analysis of RCTs to evaluate the efficacy and safety of EX versus MET in the treatment of patients with PCOS.

## Materials and Methods

### Search Strategy

Electronic databases (PubMed, Embase, Cochrane Library, CNKI, ChinaInfo, and VIP) were searched for RCTs of EX in the treatment of women with PCOS, from the time the databases were established to August 2022. The search terms used were as follows: polycystic ovary syndrome, Stein-Leventhal, ovarian degeneration, sclerocystic ovary, endocrine sexual disorders, exenatide, GLP-1, glucagon-like peptide-1, Byetta, Bydureon, AC 2993, Exendin 4. No language restrictions were applied.

Our study is registered with the International Prospective Register of Systematic Reviews (PROSPERO, CRD42022337219).

### Study Selection

#### Inclusion Criteria

Studies were included if they fulfilled the following criteria: (1) Participants: All patients with PCOS diagnosed with the Rotterdam criteria (Rotterdam ESHRE/ASRM-Sponsored PCOS Consensus Workshop Group, 2004). All patients were women of childbearing age, and with no limit in terms of country, disease course, disease degree, and whether or not PCOS was combined with a glucose metabolism disorder; (2) Intervention: EX; (3) Comparison: MET; (4) Main outcomes: pregnancy rate, ovulation rate, body mass index (BMI), homeostasis model assessment of insulin resistance (HOMA-IR), adverse events; (5) Study design: RCT.

#### Exclusion Criteria

Exclusion criteria included duplicate publications; retrospective studies; non-RCTs; non-human models; conference literature; no full-text available.

#### Data Extraction

Two authors (ZRY and SHW) independently screened the abstracts and full texts of potentially eligible articles, and extracted the data, including:

1. Title, author, and publication year;
2. Basic characteristics of the participants: number of samples, intervention measures, age, and BMI;
3. Main outcomes: pregnancy rate, ovulation rate (as determined by measuring serum progesterone levels), BMI, HOMA-IR (according to the formula: fasting insulin (µU/L) × fasting glucose (nmol/L)/22.5), adverse events;
4. Secondary outcomes: body weight, waist circumference (WC), abdominal girth (AG), waist-to-hip ratio (WHR), sex hormone-binding globulin (SHBG), serum total testosterone (TT), menstrual frequency, androstenedione (AD), dehydroepiandrosterone sulphate (DHEA-S), free androgen index (FAI), luteinizing hormone (LH), follicle-stimulating hormone (FSH), triglyceride (TG), total cholesterol (TC), high density lipoprotein cholesterol (HDL-C), low density lipoprotein cholesterol (LDL-C), hypersensitive C-reactive protein (hs-CRP), fasting plasma glucose (FPG), 2 h postprandial blood glucose (2hPBG), fasting insulin (FINS), and 2-h insulin (2hINS).

If any data were missing from a published paper, the lead author was contacted to request the data. If there were any discrepancies in the extracted data, then a third author (CQY) was consulted to resolve the differences.

### Evaluation of Study Quality

The risk of bias in each study was assessed according to Cochrane review criteria [33]. Two authors (ZRY and SHW) separately evaluated the quality of each study in seven domains: random sequence generation, allocation concealment, blinding of participants and personnel, blinding of outcome assessment, incomplete outcome data, selective reporting, and other biases, and each study was then classified as being at “low risk,” “unclear,” or “high risk” for bias. A third author (CQY) was consulted to resolve any discrepancies in classification between ZRY and SHW.

### Statistical Analysis

Statistical analysis was performed using RevMan5.4 software. The standard mean difference (SMD) or mean difference (MD) was used to evaluate continuous data, with a 95% confidence interval (CI). MD was used when continuous data were measured using the same scale. SMD was used to pool estimates from trials that measured data using different scales [34]. Dichotomous variables were assessed, and the results are expressed as relative risk (RR), with a 95% CI.

*I*^2^ and Q tests were used to analyze the heterogeneity of the studies. An *I*^2^ value > 50% or a *P* value < 0.1 indicated statistically significant heterogeneity, and these studies were then analyzed using a random-effects model. If the *I*^2^ value was still > 50%, sensitivity analyses and subgroup analyses were performed.

Z tests were performed to assess the overall effect, with a Z score of > 1.96 indicating a significant effect at a 95% value of significance.

Funnel plots were not used to present publication bias, as too few studies were included to generate such plots [33].

## Results

### Literature Search Results and Study Screening

**Fig. 1:**
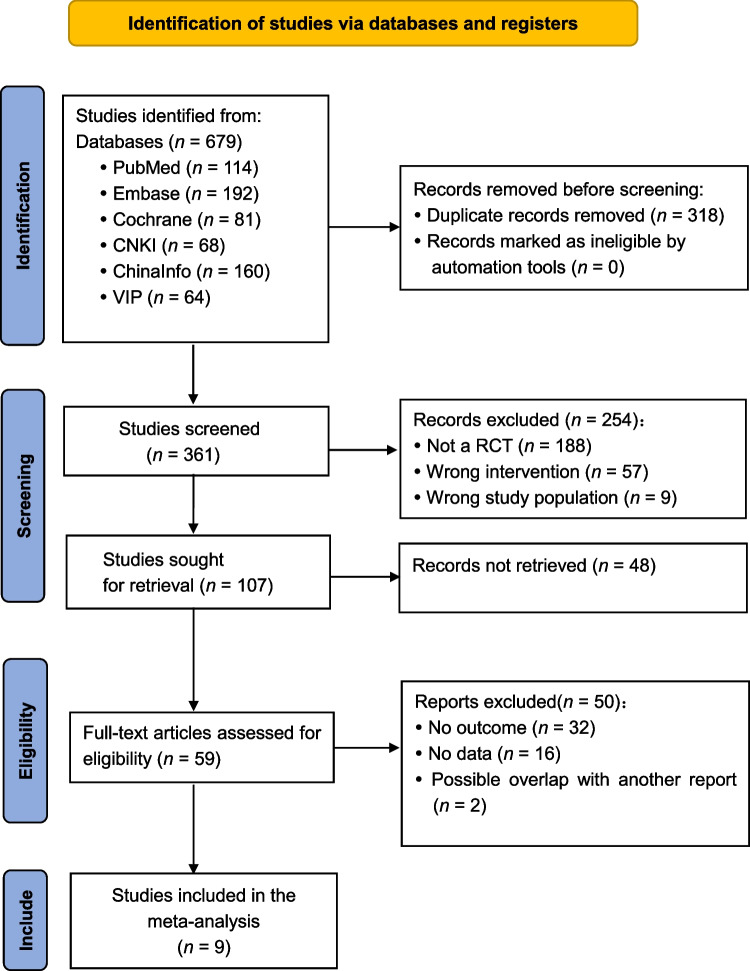
PRISMA flow diagram showing the selection of studies for inclusion. CNKI, China National Knowledge Infrastructure; VIP, China Science and Technology Journal Database; RCT, randomized controlled trial

A total of 679 articles were initially retrieved from the database searches. After duplicate articles were removed, the articles were then screened to ensure that they matched the inclusion criteria. Next, the authors of articles with missing data were contacted, and any articles for which the missing data could not be obtained were excluded. Ultimately, nine RCTs comparing EX with MET were included in the meta-analysis (Fig. 1).

### Included Studies

Nine studies comprising 785 patients were included in the meta-analysis [35–43]. For all of the included studies, the intervention group was treated with EX only (385 patients), and the control group was treated with MET only (400 patients). The patients were all women of childbearing age. The characteristics of the included studies are summarized in Table 1.

**Table 1:** Basic characteristics, objectives, intervention methods, and outcomes of the included studies

Author, year	Number of cases(T/C)	Age (years) (T/C) (mean ± SD)	BMI (kg/m^2^) (T/C) (mean ± SD)	Interventions(T/C)	Period (weeks)	Efficacy outcomes
Si et al. 2019 [35]	45/45	EX (27.98 ± 2.32)	EX (24.39 ± 2.01)	EX (5 ug BID)	25	Ovulation rate; HOMA-IR; LH; FSH; FPG; FINS
		MET (28.43 ± 2.27)	MET (24.32 ± 1.98)	MET (0.5 g TID)		
Li et al. 2017 [36]	48/64	EX (28.02 ± 2.85)	EX (28.32 ± 4.90)	EX (10 ug BID)	12	Pregnancy rate; ovulation rate; HOMA-IR; BMI; body weight; WHR; SHBG; FAI; TT; DHEA-S; menstrual frequency; AD; TC; TG; HDL-C; LDL-C; FPG; 2hPBG; FINS; 2hINS
		MET (27.13 ± 1.03)	MET (27.02 ± 3.35)	MET (1000 mg BID)		
Yuan et al. 2018 [37]	46/42	EX (30.2 ± 3.50)	EX (32.42 ± 2.03)	EX (10 ug BID)	24	HOMA-IR; BMI; body weight; WC; TG; LDL-C; FPG; 2hPBG; FINS; 2hINS
		MET (31.1 ± 2.80)	MET (31.87 ± 2.30)	MET (0.5 g TID)		
Lin et al. 2015 [38]	10/12	EX (26.7 ± 4.92)	EX (32.23 ± 2.83)	EX (10 ug BID)	12	Pregnancy rate; ovulation rate; BMI; body weight; AG; SHBG
		MET (24.0 ± 3.28)	MET (31.79 ± 2.58)	MET (0.5 g TID)		
Fan et al. 2017 [39]	38/37	EX (25.6 ± 3.20)	EX (28.64 ± 3.16)	EX (10 ug BID)	12	HOMA-IR; BMI; TT; LH; FSH; TC; TG; HDL-C; LDL-C; FPG; 2hPBG; FINS
		MET (27.6 ± 3.6)	MET (28.11 ± 4.35)	MET (0.5 g TID)		
Elkind-Hirsch et al. 2008 [40]	20/20	EX (28.2 ± 1.10)	EX (39.9 ± 1.50)	EX (10 ug BID)	24	Pregnancy rate; ovulation rate; HOMA-IR; BMI; body weight; AG; SHBG; FAI; TT; DHEA-S; menstrual frequency; TC; TG; HDL-C; LDL-C; hs-CRP
		MET (27.7 ± 1.30)	MET (41.3 ± 1.80)	MET (1000 mg BID)		
Liu et al. 2017 [41]	88/88	EX (27.93 ± 2.70)	EX (29.16 ± 3.11)	EX (10 ug BID)	12	Pregnancy rate; HOMA-IR; BMI; body weight; WC; WHR; SHBG; FAI; TT; menstrual frequency; TC; TG; HDL-C; LDL-C; hs-CRP; FPG; 2hPBG; 2hINS
		MET (27.69 ± 3.80)	MET (28.29 ± 1.86)	MET (1000 mg BID)		
Zheng et al. 2017 [42]	41/41	EX (27.7 ± 3.41)	EX (29.18 ± 4.15)	EX (10 ug BID)	12	HOMA-IR; BMI; body weight; AG; WHR; SHBG; FAI; TT; DHEA-S; menstrual frequency; LH; TC; TG; HDL-C; LDL-C; hs-CRP; FPG; 2hPBG; FINS; 2hINS
		MET (28.16 ± 3.92)	MET (29.00 ± 4.10)	MET (1000 mg BID)		
Tao et al. 2021 [43]	61/61	-	EX (30.99 ± 4.07)	EX (10–20 ug QD)	12	BMI; body weight; SHBG; FAI; TT; DHEA-S; LH; FSH; AD; TC; TG; HDL-C; LDL-C
			MET (29.64 ± 3.64)	MET (1.5–2 g QD)		

*T* treatment group, *C* control group, *EX* exenatide, *MET* metformin, *QD* quaque die/once a day, *BID* bis in die/twice a day, *TID* ter in die/three times a day, *HOMA-IR* homeostasis model assessment of insulin resistance, *BMI* body mass index, *AG* abdominal girth, *WC* waist circumference, *WHR* waist-to-hip ratio, *SHBG* sex hormone-binding globulin, *FAI* free androgen index, *TT* serum total testosterone, *DHEA-S* dehydroepiandrosterone sulphate, *LH* luteinizing hormone, *FSH* follicle stimulating hormone, *AD* androstenedione, *TC* total cholesterol, *TG* triglyceride, *HDL-C* high-density lipoprotein cholesterol, *LDL-C* low-density lipoprotein cholesterol, *hs-CRP* hypersensitive C-reactive protein, *FPG* fasting plasma glucose, *2hPBG* 2 h postprandial blood glucose, *FINS* Fasting insulin, *2hINS* 2-h insulin

### Assessment of the Risk of Study Bias

**Fig. 2:**
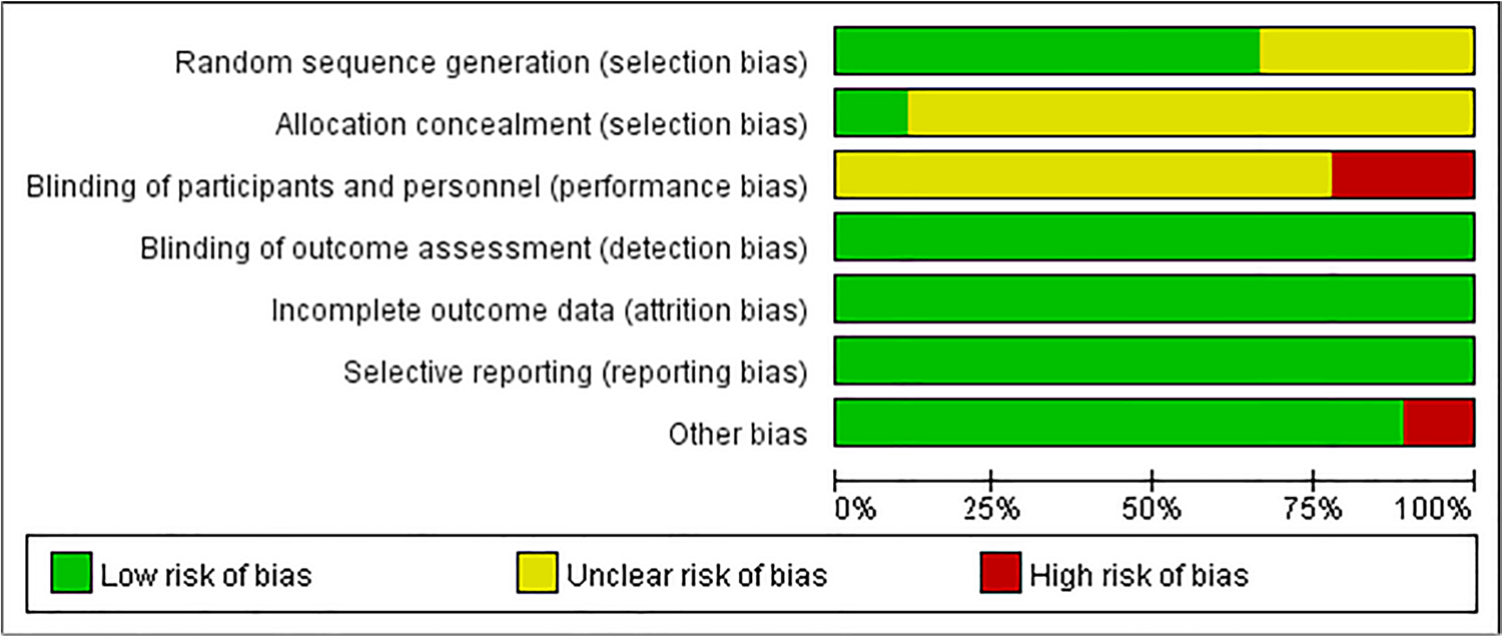
Results of the risk of bias assessment

Next, we evaluated the risk of bias in each of the nine included studies (Fig. 2 and Supp. Figure 1). Three studies [35, 39, 41] did not state clearly whether group allocation was performed by random sequence generation, whereas the other six studies did state that they used this method. Only one study [38] clearly stated that allocation concealment was applied. Two studies [38, 40] did not involve blinding of the participants and study personnel, and it was unclear if blinding was applied in the other seven studies. All studies were classified as “low risk” in terms of detection bias, attrition bias, and reporting bias. Only one study exhibited “other bias,” in that it was performed in a high-altitude geographical area.

### Meta-analysis Results

#### Primary Outcomes

**Fig. 3:**
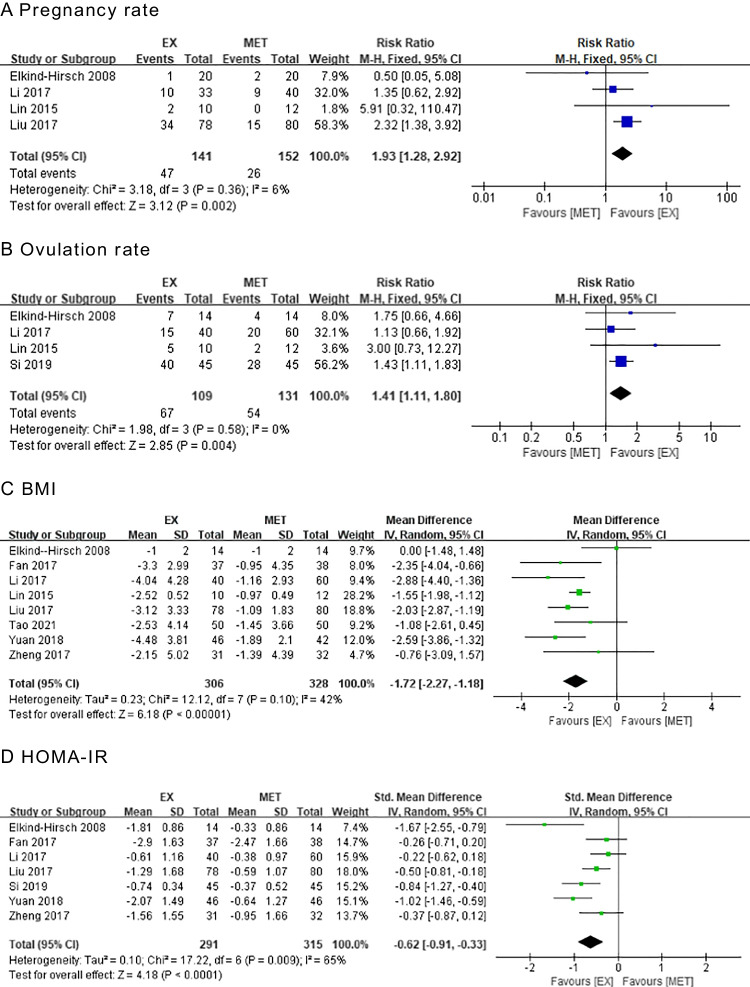
Forest plot of primary outcomes in patients treated with exenatide versus metformin, including (**A**) pregnancy rate, (**B**) ovulation rate, (**C**) body mass index, (**D**) and homeostasis model assessment of insulin resistance. CI, Confidence interval; EX, exenatide; MET, metformin

Four articles [36, 38, 40, 41] were included in the assessment of pregnancy rate. The results showed that the pregnancy rate of patients treated with EX was significantly higher than that of patients treated with MET (RR = 1.93, 95% CI 1.28 to 2.92, Z = 3.12, P = 0.002) (Fig. 3A).

Four articles [35, 36, 38, 40] were included in the assessment of ovulation rate. The results showed that the ovulation rate of patients treated with EX was significantly higher than that of patients treated with MET (RR = 1.41, 95% CI 1.11 to 1.80, Z = 2.85, P = 0.004) (Fig. 3B).

Eight articles [36–43] were included in the assessment of BMI. The results showed that the BMI of patients treated with EX was significantly lower than that of patients treated with MET (MD =  − 1.72 kg/m^2^, 95% CI − 2.27 to − 1.18, Z = 6.18, P = 0.00001) (Fig. 3C).

Seven articles [35–37, 39–42] were included in the assessment of HOMA-IR. The results showed that the HOMA-IR of PCOS patients treated with EX group was significantly lower than that of patients treated with MET (SMD =  − 0.62, 95% CI − 0.91 to − 0.33, Z = 4.18, P < 0.0001) (Fig. 3D).

### Adverse Reactions

**Fig. 4:**
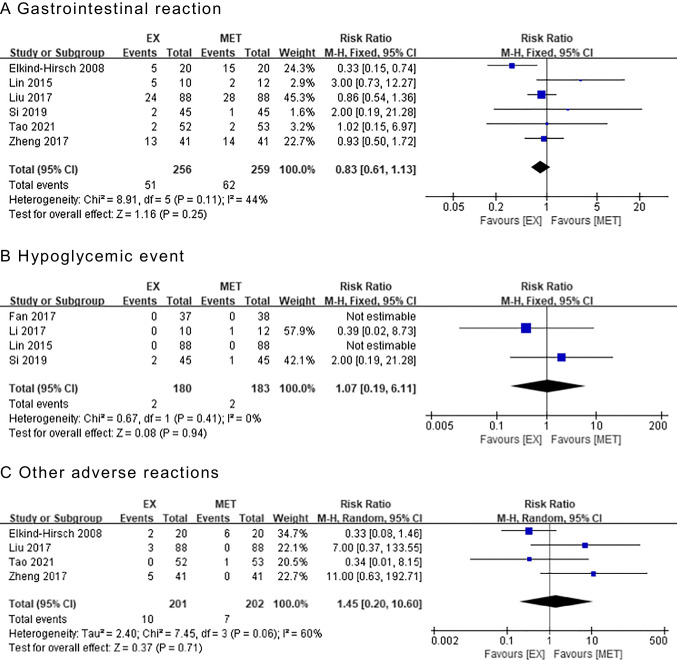
Forest plot of adverse reactions in patients treated with exenatide versus metformin, including (**A**) gastrointestinal reactions, (**B**) hypoglycemic events, (**C**) and other adverse reactions. CI, Confidence interval; EX, exenatide; MET, metformin

Six articles [35, 38, 40–43] were included in the assessment of gastrointestinal reactions (nausea, diarrhea, vomiting, etc.). The results showed that there was no significant difference in the rate of gastrointestinal reactions between the two groups (RR = 0.83, 95% CI 0.61 to 1.13, Z = 1.16, P = 0.25) (Fig. 4A).

Four articles [35, 36, 38, 39] were included in the assessment of hypoglycemic events. The results showed that there was no significant difference in the rate of hypoglycemic events between the two groups (RR = 1.07, 95% CI 0.19 to 6.11, Z = 0.08, P = 0.94) (Fig. 4B).

Four articles [40–43] were included in the assessment of other adverse reactions (headache, dizziness, fatigue, etc.). The results showed that there was no significant difference in the rate of other adverse reactions between the two groups (RR = 1.45, 95% CI 0.20 to 10.60, Z = 0.37, P = 0.71) (Fig. 4C).

Thus, there was no increase in the rate of adverse events associated with EX compared with MET.

### Subgroup Analysis

**Fig. 5:**
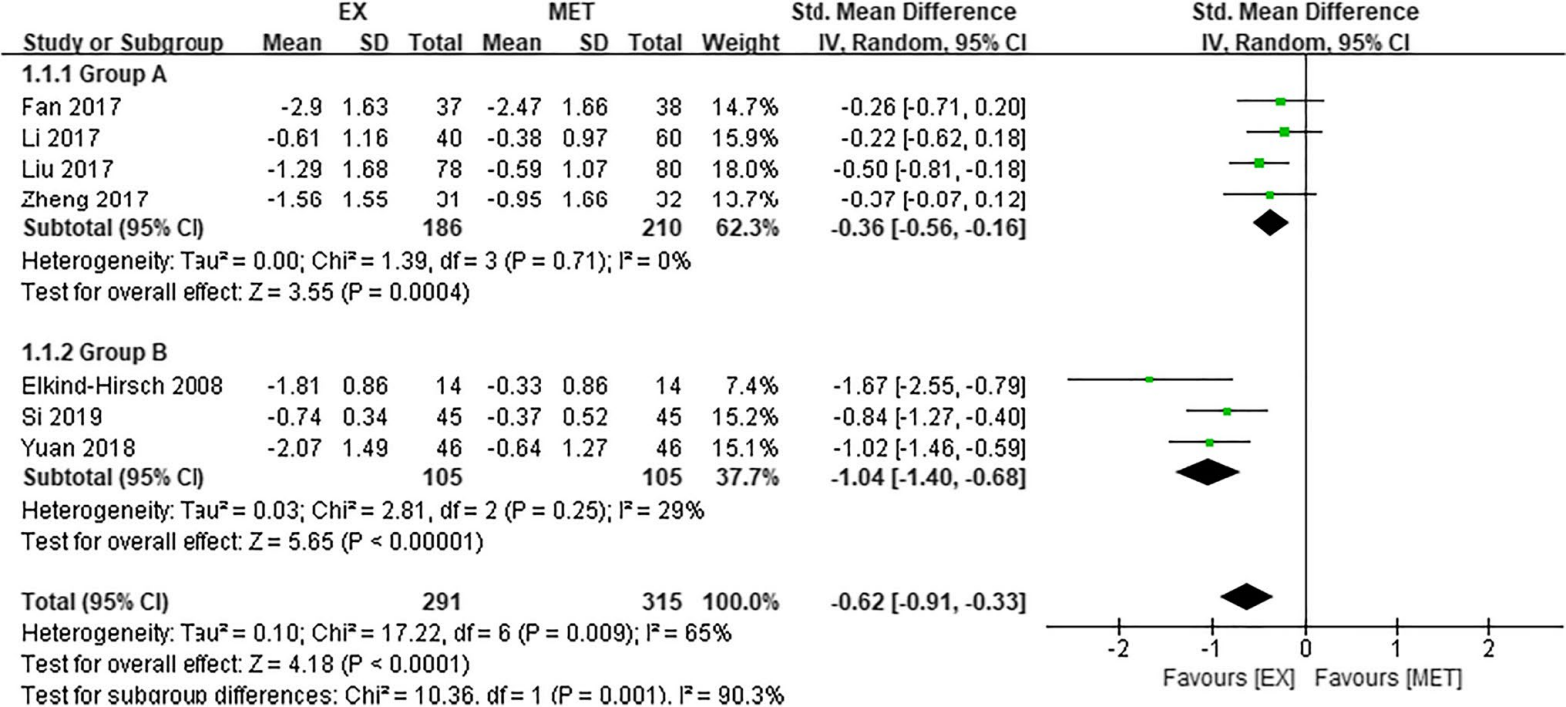
Subgroup analysis of studies evaluating homeostasis model assessment of insulin resistance in group A (less than 24 weeks) and group B (at least 24 weeks). CI, Confidence interval; EX, exenatide; MET, metformin

There was substantial heterogeneity in HOMA-IR among the seven articles included in the assessment of this outcome [35–37, 39–42]. Sensitivity analyses were performed but did not resolve the heterogeneity. In four studies [36, 39, 41, 42] the intervention lasted for 12 weeks, whereas in two studies [37, 40] it lasted for 24 weeks, and in one study [35] it lasted for 25 weeks. The studies were therefore divided into two groups based on the duration of the intervention: group A included studies that lasted less than 24 weeks, and group B included studies that lasted 24 weeks or more. Subgroup analysis showed that the source of heterogeneity was the duration of the intervention. The level of heterogeneity in each subgroup was acceptable. Further analysis showed that EX was more effective than MET at decreasing HOMA-IR in each of the two subgroups (Fig. 5).

### Secondary Outcomes

The results from the meta-analysis showed that EX was more effective than MET at reducing body weight, WC, AG, WHR, FPG, 2hPBG, FINS, 2hINS, hs-CRP, and DHEA-S. In addition, EX was more effective than MET at increasing FSH and SHBG.

There was no difference in menstrual frequency, LH, FAI, TT, AD, TC, TG, HDL-C, or LDL-C between EX and MET (Table 2).

**Table 2:** Meta-analysis of secondary outcomes

Factors	Number of participating patients (T/C)	Number of articles	Statistical method	*I*^2^ with *P* value (heterogeneity test)	Effect estimate	*P* value
Body weight	269/290	7	MD (IV, Random, 95% CI)	55% with 0.04	-2.04 (-3.48 to -0.61)	0.005
WC	124/122	2	MD (IV, Fixed, 95% CI)	47% with 0.17	-2.42 (-3.52 to -1.32)	< 0.0001
AG	53/58	3	MD (IV, Fixed, 95% CI)	1% with 0.36	-3.38 (-4.81 to -1.94)	< 0.00001
WHR	149/172	3	MD (IV, Fixed, 95% CI)	0% with 0.53	-0.04 (-0.05 to -0.02)	0.0003
SHBG	213/244	5	MD (IV, Fixed, 95% CI)	35% with 0.19	4.00 (2.33 to 5.67)	< 0.00001
TT	250/274	6	SMD (IV, Random, 95% CI)	74% with 0.002	0.10 (-0.25 to 0.46)	0.57
Menstrual frequency	163/186	4	SMD (IV, Random, 95% CI)	97% with < 0.00001	0.91 (-0.48 to 2.30)	0.20
AD	90/110	2	MD (IV, Fixed, 95% CI)	0% with 0.79	0.26 (-0.18 to 0.70)	0.25
DHEA-S	135/156	4	MD (IV, Fixed, 95% CI)	0% with 0.48	-16.47 (-27.38 to -5.56)	0.003
FAI	213/236	5	SMD (IV, Random, 95% CI)	55% with 0.07	-0.07 (-0.37 to 0.22)	0.62
LH	163/165	4	MD (IV, Random, 95% CI)	86% with < 0.0001	-0.78 (-2.03 to 0.48)	0.22
FSH	132/133	3	MD (IV, Random, 95% CI)	56% with 0.10	0.43 (0.02 to 0.84)	0.04
TG	296/316	7	SMD (IV, Random, 95% CI)	79% with < 0.0001	-0.26 (-0.62 to 0.10)	0.15
TC	250/274	6	SMD (IV, Random, 95% CI)	91% with < 0.00001	0.11 (-0.51 to 0.43)	0.73
HDL-C	250/274	6	SMD (IV, Random, 95% CI)	54% with 0.05	-0.14 (-0.40 to 0.13)	0.31
LDL-C	296/316	7	SMD (IV, Random, 95% CI)	85% with < 0.00001	-0.39 (-0.82 to 0.05)	0.08
hs-CRP	123/126	3	MD (IV, Random, 95% CI)	90% with < 0.0001	-0.48 (-0.86 to -0.09)	0.01
FPG	277/297	6	MD (IV, Random, 95% CI)	87% with < 0.00001	-0.20 (-0.40 to -0.00)	0.05
2hPBG	232/252	5	MD (IV, Random, 95% CI)	68% with 0.01	-0.36 (-0.66 to -0.07)	0.01
FINS	277/297	6	SMD (IV, Fixed, 95% CI)	0% with 0.82	-0.47 (-0.64 to -0.30)	< 0.00001
2hINS	195/214	4	SMD (IV, Fixed, 95% CI)	0% with 0.52	-0.62 (-0.82 to -0.42)	< 0.00001

*T* treatment group (exenatide), *C* control group (metformin), *SMD* standard mean difference, *MD* mean difference, *IV* inverse variance, *CI* confidence interval, *TT* serum total testosterone, *FAI* free androgen index, *FINS* fasting insulin, *2hINS* 2-h insulin, *TC* total cholesterol, *TG* triglyceride, *HDL-C* high-density lipoprotein cholesterol, *LDL-C* low-density lipoprotein cholesterol, *FSH* follicle stimulating hormone, *LH* luteinizing hormone, *SHBG* sex hormone-binding globulin, *DHEA-S* dehydroepiandrosterone sulphate, *AD* androstenedione, *AG* abdominal girth, *WC* waist circumference, *WHR* waist-to-hip ratio, *FPG* fasting plasma glucose, *2hPBG* 2 h postprandial blood glucose, *hs-CRP* hypersensitive C-reactive protein

## Discussion

The aim of this meta-analysis was to determine the reproductive efficacy and safety of EX compared with MET in patients with PCOS. We found that EX was more effective than MET in this patient population in terms of improving reproductive outcomes, promoting weight loss, and improving insulin resistance. There was no significant difference between EX and MET in terms of gastrointestinal reactions, hypoglycemia, and other adverse events.

Our meta-analysis revealed that EX is more effective than MET at improving reproductive outcomes in patients with PCOS, including pregnancy rates, ovulation rates, and sex hormone levels. In women with PCOS, ovarian follicle development is perturbed due to ovarian hyperandrogenism, hyperinsulinemia from insulin resistance, and altered intrafollicular paracrine signaling, resulting in polycystic ovarian morphology, ovulatory dysfunction, and infertility [44]. Hyperinsulinemia directly increases androgen secretion, but also increases the level of serum free testosterone by reducing the production of SHBG, which causes infertility [45]. A study performed in a DHEA-treated rat model of PCOS indicated that EX improves several aspects of follicle morphology, such as the number of cystic follicles and granule cell layers [46]. Our study showed that EX was more significantly more effective than MET at increasing SHBG and FSH and decreasing DHEA-S, although the two drugs had similar effects on other sex hormone indices (TT, LH, FAI, and AD). This suggests that the superior effectiveness of EX in this patient population in terms of improving reproductive function may be due to its effects on SHBG, FSH, and DHEA-S levels, potentially indirectly by lowering insulin resistance.

Our results also showed that EX is more effective than MET at treating features of PCOS other than reproductive function, including obesity, insulin resistance, and inflammation. Adipose tissue represents an intracrine source of androgen synthesis and may give rise to functional hyperandrogenism. EX inhibits gastric emptying mediated by the gastric vagus nerve and plays an important role in the brainstem and hypothalamic nuclei to regulate homeostatic feeding, prolong the digestive cycle, and reduce active feeding, resulting in significant weight loss [47, 48]; in keeping with this, the current meta-analysis revealed that treatment with EX resulted in significant reductions in body weight, BMI, WC, AG, and WHR compared with treatment with MET. While multiple mechanisms have been proposed for the insulin resistance seen in patients with PCOS, such as decreases in kinase activity, phosphorylation of insulin-receptor substrate, PI3K activity, and glucose transporter translocation [49, 50], impairment of downstream metabolic insulin signaling [51], and increased androgen production in the ovary [52, 53], this process is still poorly understood. Our analysis showed that EX significantly improved HOMA-IR, FPG, 2hPBG, FINS, and 2hINS compared with treatment with MET, reinforcing the importance of insulin sensitivity in these patients. Furthermore, we found that the hs-CRP level was significantly lower in patients treated with EX than in patients treated with MET, suggesting that EX could help reduce inflammation in patients with PCOS. Indeed, previous studies have shown that EX, as a GLP-1 RA, may have anti-inflammatory effects [54], and that EX can inhibit the expression of inflammatory mediators [55], although the underlying mechanism remains unclear. Taken together, these findings imply that EX is more effective than MET at treating patients with PCOS due to its enhanced ability to promote weight loss, increase insulin sensitivity, and decrease inflammation.

Interestingly, while our meta-analysis found that EX was more effective than MET in treating key symptoms of PCOS, it also showed that the adverse reactions to both drugs were comparable, as there was a similar incidence of gastrointestinal reactions, hypoglycemia, and other adverse events between the two treatment groups. A previous study showed that the main adverse reactions seen with EX are gastrointestinal reactions (usually nausea, vomiting, diarrhea, etc.), most of which resolve spontaneously without any intervention [56]. We speculate that the gastrointestinal reactions associated with EX are closely related to the regulatory effects of GLP-1 on the feeding center. Our findings suggest that EX is just as safe as MET for the treatment of PCOS, in addition to being more effective.

The main strength of our study is that pregnancy and ovulation were selected as the main outcomes to analyze the effectiveness of PCOS treatment, as these outcomes have practical significance for treatment decision-making. As an insulin sensitizer, MET is beneficial for treating metabolic abnormalities, but it is less effective at addressing problems with reproductive function; therefore, studies such as ours exploring the effectiveness of other drugs will increase the number of viable treatment options available for this condition. There were, however, some limitations to this study. First, most of the participants were overweight or obese, and therefore at higher risk for metabolic disorders, so we were unable to determine whether the beneficial effects of EX on fertility were mediated directly by its effects on the reproductive system or indirectly by promoting weight loss and improving insulin resistance; this should be investigated in future studies. Second, some of the included studies were not blinded or did not describe the blinding method, which may have biased the reliability of the results. Finally, the sample size used for the meta-analysis was relatively small; the efficacy and safety of EX should be examined in a controlled, multi-center clinical study with a larger sample size to provide stronger evidence for its use in patients with PCOS.

Given that new GLP1-RAs have been used in clinical practice, it would also be beneficial for future studies to investigate the efficacy of these new treatments compared with drugs in current use. For example, several studies have compared the efficacy of multiple GLP1-RAs, including EX, liraglutide, and semaglutide, in PCOS and found that they generally tend to promote weight loss, reduce the risk of cardiovascular disease, improve insulin sensitivity, improve hormone parameters, increase fertility, and enhance ovulation and pregnancy [27, 31, 57]. In addition, a recent review summarized the evidence for the broad cardiovascular and metabolic benefits of GLP1-RAs (lixisenatide, exenatide, liraglutide, semaglutide, albiglutide, and dulaglutide) in nondiabetic patients with a variety of conditions, including PCOS [58]. Semaglutide in particular has been shown to significantly delay gastric emptying in obese women with PCOS [59], which could have important implications for weight loss in this population. Based on our finding that EX is more effective than MET at treating key symptoms of PCOS, it is likely that some of the newer GLP1-RAs could be even more effective, and this possibility should be investigated in future studies.

## Conclusions

In summary, our meta-analysis found moderate to high quality evidence that EX is more effective than MET at improving reproductive function, and that there is no significant difference in adverse events between the two drugs. In addition, we found that the beneficial effects of EX on fertility might be related to improvements in insulin resistance and weight control. More high-quality RCTs need to be conducted to assess the long-term effects of EX, as well as the effectiveness of more potent GLP1-RAs, in patients with POCS.

## Supplementary Information

Below is the link to the electronic supplementary material.

Supplementary file1 (PNG 3925 kb)

High resolution image (TIF 609 KB)

Supplementary file2 (DOCX 32 KB)

## Data Availability

Not applicable.

## Abbreviations

AD: Androstenedione
AG: Abdominal girth
BMI: Body mass index
CI: Confidence interval
CNKI: China National Knowledge Infrastructure
DHEA-S: Dehydroepiandrosterone sulfate
EX: Exenatide
FAI: Free androgen index
FINS: Fasting insulin
FPG: Fasting plasma glucose
FSH: Follicle stimulating hormone
GLP-1 RAs: Glucagon-like peptide-1 receptor agonists
HDL-C: High density lipoprotein Cholesterol
HOMA-IR: Homeostasis model assessment of insulin resistance
hs-CRP: Hypersensitive C-reactive protein
IR: Insulin resistance
LDL-C: Low density lipoprotein cholesterol
LH: Luteinizing hormone
MD: Mean difference
MET: Metformin
PCOS: Polycystic Ovary Syndrome
RCT: Randomized controlled trial
RR: Relative risk
SHBG: Sex hormone-binding globulin
SMD: Standard mean difference
TC: Total cholesterol
TG: Triglyceride
TT: Total testosterone
VIP: China Science and Technology Journal Database
WC: Waist circumference
WHR: Waist-to-hip ratio
2hINS: 2-H insulin
2hPBG: 2-Hour postprandial blood glucose
