# Supplementary figures and images for: The Effectiveness and Safety of Exenatide Versus Metformin in Patients with Polycystic Ovary Syndrome: A Meta-Analysis of Randomized Controlled Trials
Source: Reprod Sci. 2023 Mar 31;30(8):2349–61. doi: 10.1007/s43032-023-01222-y (PMC10354168; doi:10.1007/s43032-023-01222-y)

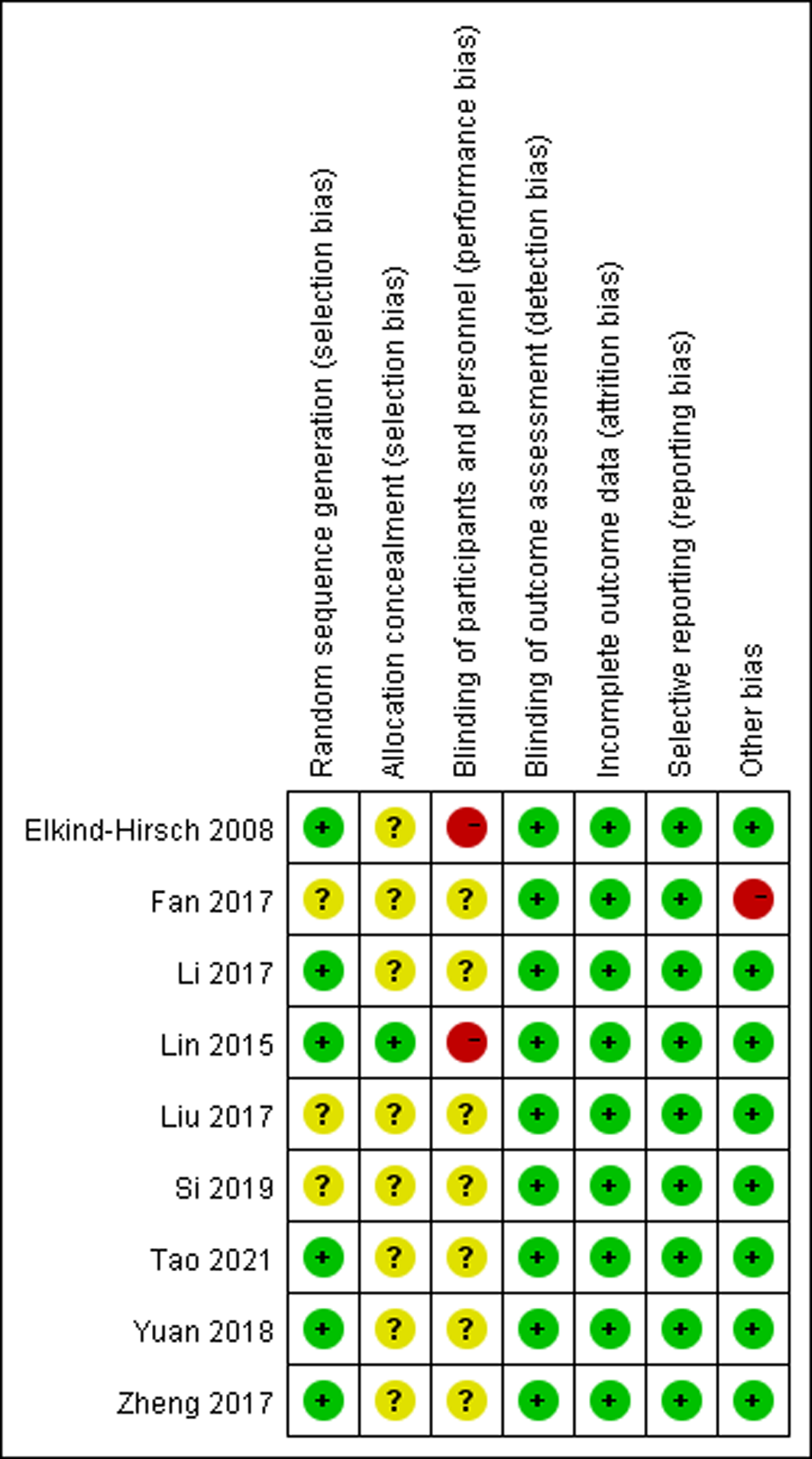

Supplement: Supplementary file 1 — Supplementary file1 (PNG 3925 kb) [file 43032_2023_1222_Fig6_ESM.png]

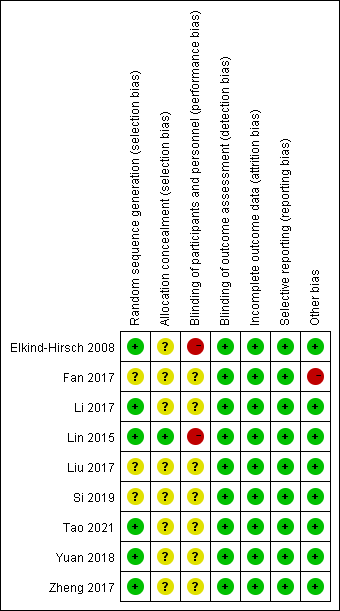

Supplement: Supplementary file 2 — High resolution image (TIF 609 KB) [file 43032_2023_1222_MOESM1_ESM.tif]
